# A Brief Overview of the Molecular Landscape of Myelodysplastic Neoplasms

**DOI:** 10.3390/curroncol31050175

**Published:** 2024-04-23

**Authors:** Rami Abdulbaki, Sheeja T. Pullarkat

**Affiliations:** Department of Pathology, Laboratory Medicine, UCLA, David Geffen School of Medicine, Los Angeles, CA 90095, USA; spullarkat@mednet.ucla.edu

**Keywords:** myelodysplastic neoplasms, myelodysplastic syndrome, next-generation sequencing, molecular features, myelodysplastic syndrome with germline predisposition syndromes

## Abstract

Myelodysplastic neoplasm (MDS) is a heterogeneous group of clonal hematological disorders that originate from the hematopoietic and progenitor cells and present with cytopenias and morphologic dysplasia with a propensity to progress to bone marrow failure or acute myeloid leukemia (AML). Genetic evolution plays a critical role in the pathogenesis, progression, and clinical outcomes of MDS. This process involves the acquisition of genetic mutations in stem cells that confer a selective growth advantage, leading to clonal expansion and the eventual development of MDS. With the advent of next-generation sequencing (NGS) assays, an increasing number of molecular aberrations have been discovered in recent years. The knowledge of molecular events in MDS has led to an improved understanding of the disease process, including the evolution of the disease and prognosis, and has paved the way for targeted therapy. The 2022 World Health Organization (WHO) Classification and the International Consensus Classification (ICC) have incorporated the molecular signature into the classification system for MDS. In addition, specific germline mutations are associated with MDS development, especially in pediatrics and young adults. This article reviews the genetic abnormalities of MDS in adults with a brief review of germline predisposition syndromes.

## 1. Introduction

Myelodysplastic neoplasm (MDS) is a clonal hematologic neoplasm characterized by persistent cytopenias (Table 1) and morphologic dysplasia. The definition of myelodysplastic neoplasms in the 2022 World Health Organization (WHO) is still consistent with the 2016 WHO edition except for replacing the term “syndrome” with “neoplasm”, as myelodysplastic neoplasms are clonal hematopoietic neoplasms [1]. One notable difference between the 2022 WHO classification and the International Consensus Classification (ICC) is the creation of a novel entity of MDS/AML in the ICC that is not in the WHO definition. Therefore, both categories should be considered by pathologists when making diagnoses and clinicians when managing patients in order to determine the most appropriate treatment options.

**Figure 1:**
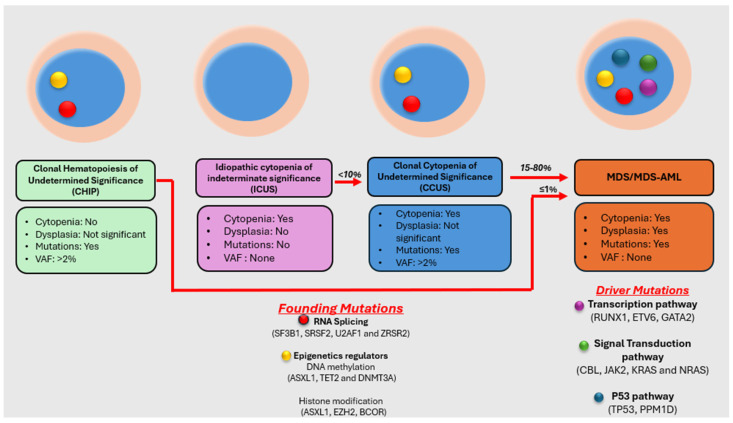
Summary of the features of CHIP, ICUS, CCUS, and MDS/MDS-AML with mutational statuses and risk of progression.

A key diagnostic challenge during the evaluation of cytopenias is differentiating MDS from other mimickers. Knowledge of the underlying genetic profile is helpful in making this distinction. The absence of driver mutations (well-defined somatic mutations known to be involved in disease pathogenesis) in cytopenias constitutes idiopathic cytopenias of undetermined significance (ICUS) with a low probability of evolving into MDS [2]. The presence of mutations in cytopenias raises the possibility of clonal cytopenias of undetermined significance (CCUS) or MDS [3]. Moreover, mutant clones without significant cytopenias can be found as an age-related phenomenon referred to as age-related clonal hematopoiesis (ARCH) or clonal hematopoiesis of undetermined potential (CHIP) [4] (Figure 1).

Dysplasia is a characteristic feature of myelodysplastic syndrome (MDS), and certain morphological findings within hematopoietic lineages are considered dysplastic. Dysgranulopoiesis refers to changes that affect the neutrophils, such as nuclear hyposegmentation (pseudo-Pelger–Huët anomaly) or hypersegmentation, as well as changes in the cytoplasm like hypogranularity. Dysplastic changes in the erythroid lineage include nuclear budding, intranuclear bridging, karyorrhexis, hypercellularity, megaloblastic changes, ringed sideroblasts, and cytoplasmic vacuoles. Dysmegakaryopoiesis is characterized by micro-megakaryocytes or non-lobed nuclei in the megakaryocytes. However, since these morphological findings can also be seen in other scenarios, such as nutritional deficiency, infections, medications (including growth factors), and bone marrow failure syndromes, the current WHO definition requires that at least 10% of cells in at least one hematopoietic lineage show dysplastic changes.

## 2. Current Classification of MDS

Morphologic dysplastic features and cytopenia have remained crucial to making MDS diagnoses in the latest WHO and ICC classification systems. MDS is categorized based on genetic abnormalities or morphology. With overwhelming evidence supporting the role of genetic events in clinical outcome, prognosis, and treatment, the 2022 ICC does not require morphological dysplasia for a diagnosis of MDS in the presence of key molecular and cytogenetics features, which include mutated *SF3B1*, del(5q), and −7/del(7q) [5].

The defining MDS genetic abnormalities that fit into both the ICC and WHO classifications include the *SF3B1* mutation, del (5q), and mutated *TP53*. Without the above genetic abnormalities and based on morphology, the WHO classifies MDS as MDS with low blasts, MDS hypoplastic, and MDS with increased blasts. The ICC recognizes entities as “MDS not otherwise specified (MDS-NOS)” and these fall into two categories: (i) MDS-NOS with at least one cytopenia and cytogenetic abnormalities such as monosomy 7 or complex karyotype even without morphologic evidence of dysplasia; (ii) MDS-NOS with single lineage dysplasia and MDS-NOS, with multilineage dysplasia, based on the morphologic features of dysplasia.

A major difference between the ICC and the WHO is in classifying cases of MDS with excess blasts ranging from 10 to 19% in the bone marrow and 10 to 19% in the peripheral blood, regardless of the presence of Auer rods. The ICC recognizes these cases as MDS/AML; this distinction is due to the fact that there is a biological flow between MDS and AML and having a 20% blast threshold to distinguish between them is not recommended, especially when they are associated and share high-risk genetic mutation profiles. This inclusion leads to more patients being enrolled in treatment regimens for AML. In addition, ICC has advocated subclassifying MDS/AML into (1) MDS/AML with myelodysplastic-related gene mutations, (2) MDS/AML-NOS, and (3) MDS/AML with mutated *TP53*. The recognition of AML/MDS as a category is supported by studies highlighting the molecular overlap between the secondary AML developed from MDS and high-risk MDS [6]. Table 2 summarizes and compares the classifications of MDS with excess/increased blasts between the WHO and ICC 2022 classifications in the absence of defining genetic abnormalities.

### 2.1. MDS with Low Blasts and Isolated 5q Deletion (MDS-5q)

Cytopenias and chromosome 5q deletion characterize this entity. The dysplastic features are striking in the megakaryocytes and are characterized by smaller cells with a single round/hypolobated nucleus. Less commonly, dysplasia in the erythroid and granulocytic lineages can also be seen; however, this does not rule out MDS-5q-. This occurs in approximately 10–15% [7] of MDS, occurs more commonly in women, and presents with macrocytic anemia and thrombocytosis. Extensive studies have been conducted on the recurrent and somatic mutations in the long arm of chromosome 5. These studies have identified a common deleted region (CDR) of approximately 1.5 Mb at 5q32–5q33.1, flanked by *D5S413* and the *GLRA1* gene, which results in the loss of several genes [8]. Other studies have shown that a partial loss of the RPS14 gene, which plays a crucial role in 18S pre-RNA processing and 40S ribosomal subunit formation, can affect erythroid differentiation through the p53 pathway. The deletion of two micro-RNAs, miR-145 and miR-146a, leads to megakaryocytic abnormalities and elevated platelets [9]. The other genetic mutation that plays a role in the pathogenesis of MDS with 5q is the casein kinase 1A1 gene (CSNK1A1), located in the common deleted region of 5q, and having a somatic mutation in CSNK1A1 on the non-deleted allele leads to haploinsufficiency and clonal dominance through the deregulated WNT/beta-catenin pathway [10]. Lenalidomide is an immunomodulatory agent and is considered one of the mainstay treatments in MDS, with a deletion in chromosome 5q. Lenalidomide has been found to be effective in reducing the need for blood transfusions [11]. Although the exact mode of action of lenalidomide remains unclear, some studies suggest that it inhibits the growth of abnormal erythroblasts by altering the expression of certain genes like *VSIG4*, *PPIC*, *SPARC*, and *PMID* [12]. This effect may be achieved by affecting the genes in the commonly deleted region (*RBM22*, *CSNK1A1*, *SPARC*, and *RPS14*) [13].

### 2.2. MDS with Biallelic TP53 Inactivation (MDS-biTP53)

p53 is a tumor suppressor protein encoded by the *TP53* gene and is considered the most frequently mutated gene in cancers [14]. This is due to the major role that p53 plays in cellular proliferation, apoptosis, DNA repair, and mediating transcription–cell death [15,16]. A loss of heterozygosity (LOH) occurs in cancer when the second wild-type allele of the *TP53* gene is lost, leading to the expression of the mutant *TP53* gene [17]. Studies have identified *TP53* alteration in 7–11% of MDS, and these alterations include mutation, deletion and copy loss of heterozygosity [18]. Transformation into AML is significantly increased in the presence of multi-hit *TP53* mutations [19,20]. Therefore, the WHO and the ICCN have adopted multi-hit *TP53* as a disease-defining entity in MDS (Table 3). The variant allele frequency (VAF) of *TP*53 in MDS has been shown to affect disease progression and prognosis. In one study, patients with MDS were divided into three groups according to their *TP53* VAF; this study showed that patients who had *TP53* VAF > 40% had inferior survival compared with patients with *TP53* VAF < 20%, concluding that VAF is an essential factor for MDS prognosis [19]. Studies have shown a significant difference when comparing the clinical outcomes of patients with monoallelic *TP53* with patients with multi-hit *TP53* status. Patients with monoallelic *TP53* status exhibit less cytopenia and lower blast percentages in the bone marrow. However, this may differ when several driver mutations co-exist [18]. Patients with complex cytogenetics and *TP53* mutation have lower overall survival than those with *TP53* WT with complex cytogenetics [21].

TP53 mutational characteristics have led to clinical trials attempting to improve treatment outcomes. To date, no treatment has been clearly shown to be superior; however, in the ASCERTAIN trial, MDS patients with bi-allelic TP53 mutations treated with the oral hypomethylating agent decitabine/cedazuridine had a favorable survival of 13 months compared with historical outcomes [22].

### 2.3. MDS with Low Blasts and SF3B1 Mutation (MDS-SF3B1)

Spliceosomes are complexes that splice and remove introns from pre-mRNA, a critical step in gene expression [23]. There are two types of spliceosomes involved in gene splicing—a U2-dependent spliceosome that removes U2-type introns and a less common U12-dependent spliceosome [24]. Splicing Factor 3b, Subunit 1 (*SF3B1*) functions as an integral component of the U2 small nuclear ribonucleoprotein complex within the spliceosome. Its role involves facilitating the splicing process of genes implicated in the pathogenesis of myelodysplastic syndrome [25,26]. Somatic *SF3B1* mutation was first reported in 34 MDS patients with defined syndromes such as ringed sideroblasts; refractory anemia with ringed sideroblasts; and refractory cytopenia with multilineage dysplasia and ringed sideroblasts [27]. The occurrence of ringed sideroblasts, coupled with an association with the *SF3B1* mutation, stems from the altered splicing of ABCB7, a mitochondrial iron transport gene, and other genes involved in mitochondrial metabolism These alterations lead to ineffective erythropoiesis and the development of ringed sideroblasts [27,28]. MDS-*SF3B1* has replaced MDS with ringed sideroblasts in the WHO 4th Edition classification. The diagnostic criteria for MDS-SF3B1 include the presence of the *SF3B1* mutation with a variant allele frequency (VAF) of at least 10% and the absence of multi-hit *TP53* or *RUNX1* mutations, 5q deletion, monosomy 7, or complex karyotype [1,20]. Patients with the *SF3B1* gene mutation in the absence of certain co-mutations have favorable prognoses [29,30].

## 3. Genetic Alterations in MDS

The evolution of MDS has been well-studied, and the model of initiation, progression, and transformation is a multistep event in MDS [31]. Recurrent mutations are commonly seen in 40–50 genes associated with MDS, and they are included in most commercially available NGS panels. Almost all patients with MDS have at least one somatic mutation [32], and the mutated gene pattern reveals biological pathological mechanisms involved in MDS. The dominant share of mutations in MDS involves proteins crucial for regulating the epigenetic aspects of gene expression. This encompasses DNA methylation control (*TET2*, *DNMT3A*, *IDH1*, and *IDH2*) and histone modification (*ASXL1* and *EZH2*). RNA splicing genes other than *SF3B1* also play a role in MDS pathogenies (*SRSF2*, *U2AF1*, and *ZRSR2*) [32] (Table 4). Studies have shown that mutations in the spliceosome and the methylation pathways with additional mutations in *RUNX1*, *BCOR*, *EZH2*, *CBL*, *CUX1*, and *IDH1/IDH2* are highly specific for MDS in the right clinical setting [33]. Mutation patterns featuring the *SF3B1* or *TP53* mutations, along with alterations in *DNMT3A*, *TET2*, and *ASXL1* (DTA genes) co-occurring with additional mutations, consistently exhibit a strong correlation with overt dysplasia when the variant allele frequency (VAF) is below 0.20. This implies that these genetic irregularities contribute to the manifestation of an overt dysplastic phenotype early in their developmental trajectory [33]. In contrast, other genotypes, including isolated or multiple mutations in DTA genes and patterns involving SRSF2, display low clinical expressivity and necessitate complete dominance to give rise to overt dysplasia in a clinical setting [33] Less commonly mutated genes include those involved in signal transduction and transcription factors (Figure 1). The impact of mutational VAF in MDS and its association with prognosis has shown to be different between the specific genes and the co-mutated genes present; for example, in one study, the *NRAS* gene was associated with leukemia transformation regardless of whether it had low or high VAF [34]; in another study, co-mutation of *EZH2* with *NRAS* mutation was associated with poor overall survival regardless of the mutational burden of *NRAS* [35]. However, high allele burden in genes like *TET2*, *TP53*, and *SF3B1* has an impact on survival and treatment response [35].

## 4. Prognostication of MDS Based on Genetic Signature

Molecular genetic testing can help assess the prognosis of MDS patients. Mutations in MDS are divided into two types by some study groups. Type 1, associated with faster progression to secondary AML, includes alterations in *FLT3*, *PTPN11*, *WT1*, *IDH1*, *NPM1*, *IDH2*, and *NRAS*. Type 2, with alterations in *TP53*, *GATA2*, *KRAS*, *RUNX1*, *STAG2*, *ASXL1*, *ZRSR2*, and *TET2*, is rich in high-risk MDS [14]. A diversity of mutations and increased clone sizes have been described during disease progression, from low-grade to high-grade MDS [31,36]. Mutations in *SF3B1* indicate a favorable prognosis, while mutations in *TP53*, *RUNX1*, *EZH2*, and *NRAS* are associated with poor outcomes [37]. Mutations in *DDX41* have been linked to an increased percentage of blasts and a higher risk of developing acute myeloid leukemia (AML) in individuals with myelodysplastic syndrome with excess blasts. However, these mutations are associated with a favorable overall survival rate [38]. On the other hand, in multi-hit *TP53*, mutations in *FLT3* (both TKD and ITD) and partial tandem duplication of *KMT2A* have been identified as predictors of adverse outcomes and an increased risk of transformation into AML [39]. A 2018 study by the Working Group for the MDS Molecular Prognostic Committee on 339 MDS patients found that 55% of those with a complex karyotype had *TP53* mutations, a greater determinant of poorer overall survival than either monosomy or a complex karyotype.

The risk stratification for MDS used in the past was based on Revised International Prognostic Scoring System [IPSS-R], which considered hematologic parameters and cytogenetic abnormalities. However, since the mutational profile has a significant impact, this has led to the development of the revised IPSS-Molecular [IPSS-M]. IPSS-M includes molecular data, which are a valuable tool for making clinical decisions.

### 4.1. MDS with Somatic Mutations Involving DNA Methylation

*DNMT3A* mutations have been identified in 8% of patients with MDS and in about 22% of cases in de novo AML [40]. Specifically, the presence of *DNMT3A R882* mutations in MDS is correlated with leukopenia and associated with mutations in *SRSF2* and *IDH2*, with characteristics such as an excess of blasts and an elevated likelihood of transforming into AML, particularly when compared with cases lacking *DNMT3A R882* mutations [41].

The tet methylcytosine dioxygenase 2 gene (TET2) has a significant role in promoting the self-renewal of stem cells, and its frequency in MDS is about 19% [42]. Patients with MDS who have more than one *TET2* mutation have significantly increased monocyte counts in the bone marrow and peripheral blood, more specifically *TET2^I1873T^* mutation has been found to be significantly associated with progression to chronic myelomonocytic leukemia (CMML) [43]. In addition, the *TET2* mutation is frequently the first hit mutation in CMML, and having a second *TET2* might be a driver to the full CMML disease phenotype [44,45]. Isocitrate dehydrogenase 1/2 (IDH1/2) genes have been identified in cases of AML and MPN and have been detected in 5% of MDS cases. Some studies have shown that the *IDH1* mutation is associated with poor prognosis and adverse effects, in contrast to the *IDH2^R140Q^* mutation, which does not play a role in leukemia-free or overall survival [46,47].

### 4.2. MDS with Somatic Mutations Involving Histone Modifications

*ASXL1* mutations exclusively occur within exon 12 of the gene and are thought to result in the truncation of plant homeodomain10 at the protein’s C-terminus, and this truncation affects its involvement in chromatin modification [48]. In MDS, truncating mutations in *ASXL1* are identified at an overall incidence of 15–25% and are independently associated with a poor prognosis [49]. When the *ASXL1* mutation frequency was compared with karyotype findings, it was more commonly associated with del(7q)/monosomy 7 and uncommon in del (5q)/monosomy 5 karyotypes [50]; in addition, some groups have suggested using it as a molecular marker for minimal residual disease and disease progression [50]. From a treatment standpoint, mutations in *ASXL1* have been reported to adversely affect responses to hypomethylating agents and lenalidomide but not the response to erythropoiesis-stimulating agents [51].

*EZH2*, also known as enhancer of zeste 2 polycomb repressive complex 2 subunits, functions as the catalytic subunit of the polycomb repressive complex 2, and is involved in repression of several tumor suppressor genes [52,53]. Nonsense and frameshift mutations in *EZH2* occur in 5–10% of MDS. *EZH2* alterations are independently associated with poor prognosis in MDS [54].

### 4.3. MDS with Somatic Mutations Involving RNA Splicing

Serine/arginine-rich splicing factor 2 (SRSF2) is a member of the SR protein family that plays a role in pre-mRNA splicing and spliceosome activities [55]. SRSF2 is one of the most frequently mutated genes in MDS [56] and is strongly associated with male sex and older age. The frequency of the mutation is similar in patients with low-risk MDS and high-risk MDS. It is frequently associated with additional mutations, especially *RUNX1*, *ASXL1*, and *IDH2* which may explain the inferior overall survival [57].

U2 small nuclear RNA auxiliary factor 1 (*U2AF1*) is a gene that encodes for the auxiliary U2 pre-mRNA splicing complex, and its genetic alteration can lead to pre-mRNA splicing hematopoiesis alterations in primary progenitor cells [58]. Mutated *U2AF1* is associated with shorter overall survival in MDS and are more likely to transform to AML compared to unmutated *U2AF1*, suggesting that these patients should undergo a more aggressive treatment plan when this mutation is present [59].

### 4.4. MDS with Somatic Mutations Involving p53 Pathway

*PPM1D* is frequently recurrent in therapy-related MDS, arising in patients with a history of cytotoxic chemotherapy and radiation exposure, and the frequency of *PPM1D* mutations alone is 51% in therapy-related MDS compared with *TP53* mutations alone (39%) or concurrent *PPM1D* and *TP53* mutations (54%) [60].

## 5. Non-Coding RNA in MDS

The regulation of gene expression is controlled by non-coding RNAs such as microRNAs (miRNAs) and long non-coding RNAs (lncRNAs). Their role in normal hematopoiesis involves the regulation of the early stages of hematopoiesis, which is identified by the expression of specific miRNAs such as miR-181, miR-223, and miR-142 [61]. The InRNAs are expressed in stage- and lineage-defining patterns from the early hematopoietic progenitor cells to the mature cells [62]. Since they play a role in erythropoiesis and megakaryocytopoiesis, the downregulation of miR-150 and miR-145 has been associated with MDS [63,64]. In addition, some studies have suggested using miRNAs as a potential prognostic marker; for example, the overexpression of miR-125a has been shown to be associated with poor prognosis [65]. Regarding the role of lncRNAs in MDS, the expression of the lncRNAs KCNQ10T1 and HOXB-AS3 is associated with poor prognosis [66,67].

## 6. Genetic Germline Predisposition in Myelodysplastic Neoplasms

It is sometimes observed that certain variants detected on NGS panels are germline in nature, rather than somatic. This is especially true for those variants with a VAF near 50%. Genetic germline mutations associated with various hematological neoplasms, including acute myeloid leukemia (AML), myelodysplastic neoplasms (MDS), myeloproliferative neoplasms (MPN), and MDS/MPN, are well described in the 2022 WHO classification. Myeloid neoplasm with the germline *DDX41* mutation has been recognized as a cause of MDS in adults [68], with hematological features that include leukopenia and erythroid dysplasia with hypocellular bone marrow with increased blast counts and acute myeloid leukemia. The most common mutations associated with patients with germline *DDX41* mutations are *TP53*, *ASXL1*, *SRSF2*, and *DNMT3A*, with no significant difference in the incidence of these mutations between the germline and somatic DDX41 variants [69]. The germline mutation of RUNX1 is associated with familial platelet disorders with predispositions to myeloid malignancy (FPDMM), which is an autosomal-dominant disease characterized by thrombocytopenia and platelet dysfunction that leads to an increased risk of hematological malignancy, such as myelodysplastic syndrome [70]. Differentiating between the somatic and germline variants holds significance, influencing the choice of an appropriate allogeneic transplant donor, monitoring patients for non-hematologic complications like epithelial cancers in Li–Fraumeni syndrome, and facilitating family counseling. To discern between germline and somatic variants, it might be essential to have a culture of fibroblasts or employ another non-hematopoietic cell source devoid of blood contamination. A detailed overview of germline syndromes is beyond the scope of this review.

## 7. Conclusions

Comprehending the genetic evolution of MDS is essential for accurate diagnosis, prognosis, and effective treatment. Negative molecular test results can significantly impact diagnostic evaluations given the elevated negative predictive value associated with a normal result for MDS. Molecular profiling has become integral to managing MDS, helping to stratify patients into different risk categories and guiding treatment options. Novel therapeutic strategies targeting specific genetic alterations offer hope for more personalized treatment options for MDS patients. It is important to consider certain limitations when it comes to molecular testing in MDS. For instance, in a patient suspected of having MDS, certain variants may be detectable that are not necessarily present in myeloid cells. If both a B-cell clonal population and dysplastic myeloid cells coexist in the same patient, an SF3B1 variant could be found in either the B-cell clone or the MDS. The accurate determination of this scenario typically requires mutation testing of lineage-sorted cells, a test not commonly available in clinical settings. Clinical NGS assays conducted in both commercial and academic institution laboratories often entail a significant billing markup, thereby introducing substantial expenses to the patient’s assessment. The accessibility of molecular testing remains limited, particularly in developing nations, and the sensitivity and gene coverage of available testing panels can vary.

Lastly, NGS testing is sometimes ordered without an appropriate indication due to the lack of standard recommendations on the timing of these studies.

Clinicians exhibit differing degrees of comfort and proficiency in deciphering cancer genome tests. Thus, maintaining continuous education becomes imperative, and pathologists play a pivotal role in aiding clinicians by offering clear and updated variant interpretations in their reports.

## Figures and Tables

**Table 1:** Defining cytopenia values in MDS.

Cytopenia Type	Reference Values
Anemia	Hb < 13 g/dL in males Hb < 12 g/dL in females
Leukopenia	Absolute neutrophil count < 1.8 × 10^9^/L for leukopenia
Thrombocytopenia	Platelets < 150 × 10^9^/L

**Table 2:** Classifications of MDS according to blast percentage in the bone marrow and peripheral blood in the absence of defining genetic abnormalities in the WHO 5th Edition and ICC 2022 classifications.

Blast Percentage in MDS	MDS Classifications: WHO 5th Edition	MDS Classifications: ICC 2022
Bone marrow, <5% Peripheral blood, <2%	Myelodysplastic neoplasm with low blasts	Myelodysplastic syndrome–NOS
Bone marrow, 5–9% Peripheral blood, 2–4%	MDS with increased blasts-1 (MDS-IB1)	MDS with excess blasts (MDS-EB)
Peripheral blood, 5–9%	MDS with increased blasts-2 (MDS-IB2)	MDS with excess blasts
Peripheral blood, 10–19% Bone marrow, 10–19%	MDS with increased blasts-2 (MDS-IB2)	AML/MDS

**Table 3:** The molecular diagnostic criteria for MDS-mutated *TP*53 in the ICC 2022 classification.

**MDS with Multi-hit *TP53*—WHO 2022**
Proof of one or more pathogenic *TP53* sequence variations (exon 4-11). If only one *TP53* alteration is detected, variant allele frequency should exceed 49%, as evidenced for * LOH by deletion (cytogenetics) or ** CNLOH.
**MDS-Mutated *TP53*—ICC 2022**
Two or more *TP53* mutations (each with VAF ≥ 10%) or a single *TP53* mutation with VAF > 50% and/or VAF ≥ 10% with LOH at the 17p deletion. Single TP53 mutation with VAF 10–49% with a complex karyotype and/or 17p deletion.

* LOH: Loss of heterozygosity, ** CNLOH: copy neutral loss of heterozygosity.

**Table 4:** Somatic mutations seen in MDS.

Epigenetic regulators via DNA methylation	*TET2*, *DNMT3A*, and *IDH1/2*
Epigenetic regulators via histone modification	*ASXL1*, *EZH2*, and *BCOR*
Transcription pathways	*RUNX1*, *ETV6*, and *GATA2*
Signal transduction pathway	*CBL1*, *JAK2*, *KRAS*, and *NRAS*
RNA splicing	*SF3B1*, *SRSF2*, *U2AF1*, and *ZRSR2*
*P53* pathway	*PMM1D*
